# Antidermatophytic Activity of *Pogostemon parviflorus* Benth

**Published:** 2010

**Authors:** Batool Sadeghi-Nejad, Subhash Sadhu Deokule

**Affiliations:** a *Department of Mycoparasitology, Medical School, Ahvaz Joundi-Shapour University of Medical Sciences, Ahvaz, Iran.*; b * Department of Botany, University of Pune Ganeshkhind, Pune, India*.

**Keywords:** Phytochemical, *Pogostemon parviflorus*, Antidermatophytic activity, HPTLC studies, Triterpenes

## Abstract

In the developing countries of tropical regions, mycotic infections are common cause of skin diseases.The use of medicinal plants in the treatment of skin diseases including mycotic infections is an age-old practice in many parts of the world. The drugs used against dermatophytosis have several side effects, but limited efficacy. There is therefore a distinct need for discovery of new, safer and more effective antifungal agents. Medicinal plants used in traditional folk medicine may help us to overcome the growing problem of resistance to antifungal drugs and also their relative toxicity. In this study, in vitro antifungal activity of *Pogostemon parviflorus *leaf extracts were evaluated against three different genera of dermatophytes including *Microsporum*, *Trichophyton *and *Epidermophyton*, using the agar dilution method. *Pogostemon parviflorus *Benth. belongs to Labiatae family. The ethanolic extract of *Pogostemon parviflorus *leaf inhibited the growth of tested dermatophytes at different concentrations. The ethanolic extract of *Pogostemon parviflorus *leaf completely prevented the growth of tested dermatophytic species, with minimum inhibitory concentration (MIC) values between 2.5-10 mg/mL. The minimum fungicidal concentration (MFC) values of this plant were also in the range of 2.5-10 mg/mL. Results of phytochemical screening tests indicated that the leaf of *Pogostemon parviflorus *contained saponins, reducing sugars, tannins, phenols and proteins, but it did not have any glycosides, anthraquinones, alkaloids or flavonoids. Results of High Performance Thin Layer Chromatography (HPTLC) studies indicated that the ethyl acetate extract of *Pogostemon parviflorus *leaves included triterpenes, as 10 and 14 peaks of ultra violet (UV) absorption were observed in 254 nm and 366 nm, respectively. Hence, triterpenes may be responsible for antidermatophytic activity of this plant.

## Introduction

**Figure 1:**
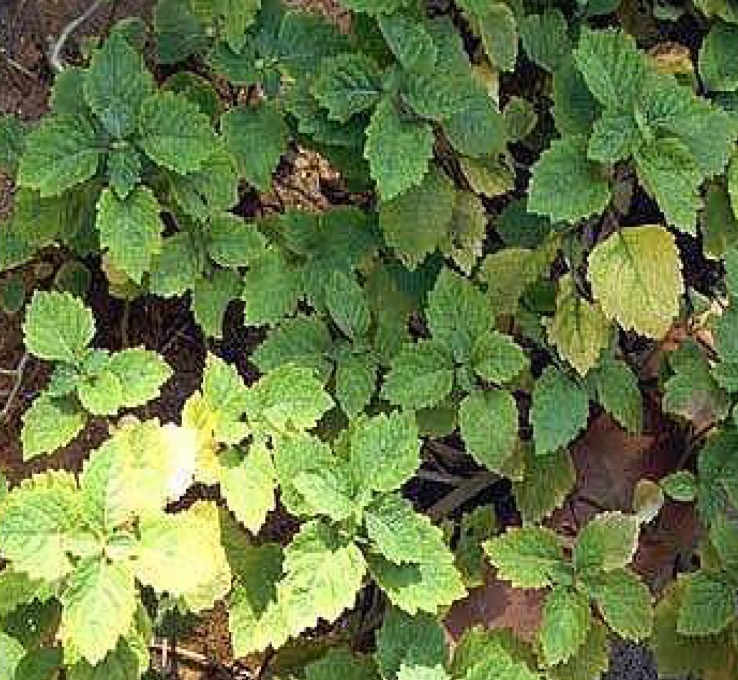
*Pogostemon parviflorus *Habitat

**Figure 2:**
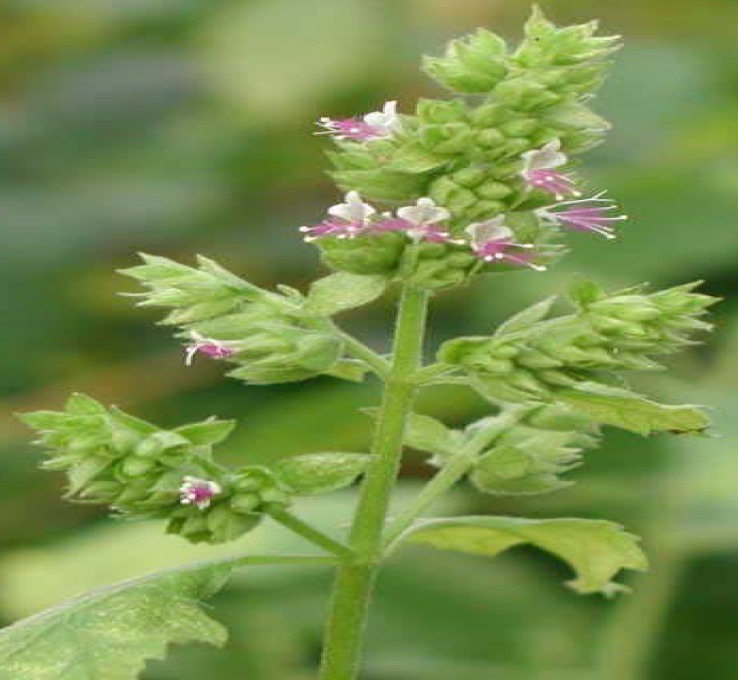
*Pogostemon parviflorus *Flowering twig

*Pogostemon parviflorus *has a strong odor. It grows in areas with high annual rainfall (Figures 1 and 2). This plant has antiseptic activity and it is useful in the treatment of enteritis, eczema and mycotic enteritis (1). Due to the increasing number of immunocompromised individuals, fungal infections have increased in the last two decades (2), among which skin fungal infections are very difficult to eradicate (3). Dermatophytes produce a variety of problems, such as Athlete’s foot and nail infections which lead to debilitation of the patients, and they can also spread to other areas of the body and to other individuals (4). Human mycoses are not always successfully treated because of their resistance to antifungal drugs, or ineffectiveness and side effects of these agents. Hence, it is of urgent importance to search for more effective and less toxic new antifungal agents through their detection in medicinal plants. Furthermore, new antifungal agents are still needed to improve the treatment of superficial fungal infections (5, 6).

## Experimental

*Plant material*

*Pogostemon parviflorus *was collected from Mulshi district, Pune Maharashtra state, India. 

This plant specimen was collected in flowering and fruiting state and identified by the botanical survey of India. A voucher specimen has been deposited at the herbarium of Botany department of Pune University, India. The healthy and disease-free leaves were separated and shade dried to avoid decomposition of chemical constituents. Leaves were powdered in grinder and stored in clean and dry airtight containers for further studies.

*Preparation of plant extracts*

The leaves of *Pogostemon parviflorus *were extracted in ethanol. To 10 g of each powdered material was added 100 mL ethanol 80% (drug/solvent ratio of 1:10 w/v) in a conical flask for maceration.Flask was then plugged with cotton and placed on a rotary shaker at 190-220 rpm for 72 h at room temperature (7). Finally, the suspension was filtrated through a Buckner funnel and Whatman filter paper #1. 

The ethanolic extract was evaporated to dryness in an oven or in a water bath at 45°C. One gram of the dried extract was then dissolved in 1 mL 100 % dimethyl sulfoxide (DMSO). The final concentration of each extract was adjusted to 1000 mg/mL. 

*Dermatophyte isolates*

For the evaluation of antifungal activity, 3 strains obtained from the Persian Type Culture Collection (PTCC), Tehran, Iran, including *Trichophyton mentagrophytes *PTCC 5054, *Microsporum canis *PTCC 5069, *M. gypseum *PTCC 5070; 13 as well as other strains isolated from different lesions of patients in a clinical laboratory in Ahwaz, Iran, including *Microsporum canis *(n = 2): MC-1, MC-2*, M. gypseum *(n = 3): MG-1, MG-2, MG-3, *Trichophyton rubrum *(n = 2): TR-1, TR-2, *T*. *mentagrophytes *(n = 3): TM-1*, *TM-2, TM-3 and *Epidermophyton floccosum *(n = 3): EF-1, EF-2, EF-3 and identified by standard procedures (8). Sabouraud dextrose agar (SDA) at 25°C was used to maintain isolates. For antifungal assay, each dermatophyte isolate was subcultured onto sabouraud dextrose agar (Hi-Media, India) slants and incubated at 28-30°C for 4-5 days and subcultured every 15 days to prevent pleomorphic transformations (7). 

*Preparation of fungal inoculum*

A standardized inoculum was prepared by counting the microconidia, microscopically. For this purpose, the suspension of conidia was prepared using sterile distilled water or 0.85% physiological saline solution. The dispersing fluid was added to the slant tube culture and the surface of culture was gently rubbed by a sterile bent glass rod to dislodge the conidia from the hyphal mat. The suspension was then transferred to a sterile centrifuge tube and the volume was adjusted to 5-10 mL with sterile saline. The final suspension of conidia was counted with a hemocytometer cell counting chamber. The inoculum of cell or spore suspensions were prepared, as described elsewhere (9, 10) , and adjusted to 104-105 colony-forming units (CFU) per mL. 

*Antifungal susceptibility testing*

The fungistatic activity of different extracts was evaluated by the agar dilution method (7, 11, 12). One thousand milligrams of the ethanolic extract was dissolved in 1 mL of sterile DMSO, serving as the stock solution (7). For the assay method, the stock solution of extract was two-fold diluted with sterile distilled water or saline solution to produce serial decreasing dilutions ranging from 0.078-20 mg/mL. Then 5 mL Mycosel agar medium was dispensed in each petri dish (60 mm in diameter), under laminar flow (aseptic condition), and cooled to 45°C. Into the non-solidified media 100 μL of the extract stock solution plus 50 μL of the dermatophyte suspension (10^5^ CUF/mL) removed from a seven days old culture of fungi, was added, and evenly mixed. The plates were then incubated at 28-30°C. 

MICs were visually recorded, based on the control fungus growth, up to 15 days for dermatophytic species. The antifungal agents like griseofulvin (Sigma) and Ketoconazole (Janssen Pharmaceutical) were used as the positive controls. Drug-free solution (only containing an appropriate amount of DMSO) was also used as the blank control for verification of fungal growth. MIC value was defined as the lowest extract concentration capable of inhibiting fungal growth, and MFC value was defined as the lowest extract concentrations showing no visible fungal growth after the incubation time. MIC_50_ and MIC_90_ values were the lowest extract concentrations at which 50% and 90% of the clinical isolates were inhibited (13). Dermatophyte plates were examined visually for 50% and 90% growth inhibition, compared to the growth control. MIC results were recorded in μg/mL. Every strain was tested in triplicate and a new inoculum was prepared for each assay. Duplicate plates were used for each assay. 

*Phytochemical study*

The leaf of *Pogostemon parviflorus *was evaluated qualitatively for the presence of saponins, reducing sugars, tannins, alkaloids, proteins, glycosides, anthraquinones and flavonoids. In the present investigation, the ethyl acetate extract of *Pogostemon parviflorus *leaf was subjected to TLC, using a precoated silica gel F254 plate. A solvent system of acetone . ethyl acetate . petroleum ether (0.5: 0.5:2.0) [AEP] was used for obtaining the best resolution for spots. HPTLC fingerprint for the same extract was obtained at 254 nm and 366 nm.

*Statistical analysis*

Data analysis was performed, using the SPSS program version 10 (SPSS Inc., USA). Analysis of variance was conducted, using the general one-way ANOVA with post hoc comparison of mean values by LSD.

## Results and Discussion

**Figure 3:**
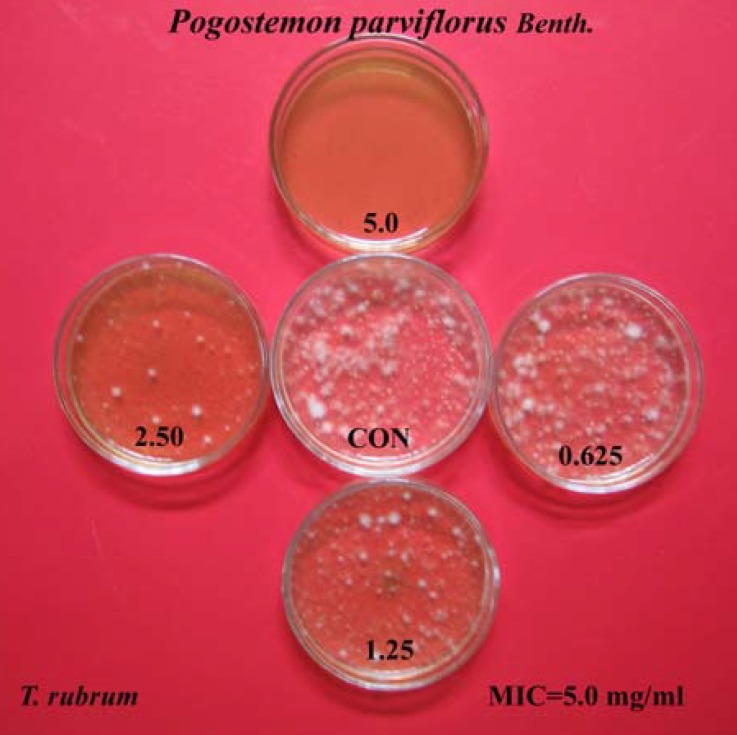
Inhibitory effects of ethanolic extract of *Pogostemon parviflorus *leaf on the growth of *T. rubrum *by agar Dilution method on Mycosel medium. The decreasing dilutions used, ranged from 0.078-5.0 mg/mL

The ethanolic extract of *Pogostemon parviflorus *leaf completely prevented the growth of tested dermatophytic species, with MIC values between 2.5-10 mg/mL. MIC_90_ and MIC_50_ values were 1.250-5.000 and 0.312-1.250 mg/mL, respectively. The lowest MIC_90_s and MIC_50_s were concerned with the species of *T. mentagrophytes *and the highest MIC_90_s and MIC_50_s were observed with the strains of *M. canis*. The MFC values of this plant were also in the range of 2.5-10 mg/mL. Finally, the *T. mentagrophytes *species were found to be more sensitive than the other dermatophytic species, while species of *M. canis *were the most resistant among the five tested dermatophytic species, against inhibitory effects of *Pogostemon parviflorus. *The results have been shown in Tables 1 and 2, as well as Figure 3. 

**Table 1:** MICs (mg/mL) of *Pogostemon parviflorus *leaf extract, compared to griseofulvin and Ketoconazole.

**Test agent **	**MIC value (mg/mL)** ^a^					
	**Mc** ^b^	**Mg** ^b^	**Ef** ^b^	**Tr** ^b^	**Tm** ^b^
*Pogostemon parviflorus *	10.00	5.00	5.00	5.00	2.500	
Griseofulvinc	12.5	100	25	50	100	
Ketoconazolec	25.00	6.25	0.78	25.00	6.25	

^a^ Values are given as mean (mg/mL) of triplicate experiments.

^b^ Mc: *Microsporum canis *PTCC 5069; Mg: *Microsporum gypseum *PTCC 5070; Ef: *Epidermophyton floccosunm *EF-3; Tr: *Trichophyton rubrum *TR-1; Tm: *Trichophyton mentagrophytes *PTCC 5054*. *

^c^ Griseofulvin and Ketoconazole served as positive control.

**Table 2:** Minimum inhibitory concentration (MIC) and minimum fungicidal concentration (MFC) of *Pogostemon parviflorus *leaf extract, griseofulvin and Ketoconazole against dermatophytes, using the agar dilution method

**Dermatophytes ** **(number of strains) **	**Antifungal Compounds **	**MIC ** ^a^ ** and MFC**				
		**Range **	**50%b **	**90%b **	**MFC **	**Geometric mean MIC **
*T. mentagrophytes *(3) *T. menta. *PTCC5054	KTZ GRS PPL	0.78-6.25 12.5-100 0.156- 1.250	1.56 25 0.312	6.25 100 1.250	12.5 200 2.500	3.52 56.25 0.703
*M. gypseum *(3) *M. gypseum *PTCC5070	KTZ GRS PPL	0. 78-6.25 12.5-100 0.312- 2.500	1.56 25 0.625	6.25 100 2.500	12.5 200 5.000	3.52 56.25 1.406
*M. canis *(3) *M. canis *PTCC5069	KTZ GRS PPL	1.56- 12.50 3.12-25.00 0.625- 5.000	3.12 6.25 1.250	12.5 25 5.000	25 50 10.00	7.03 14.06 2.513
*T. rubrum *(2)	KTZ GRS PPL	3.12-25.00 6.25- 50.00 0.312- 2.500	6.25 12.5 0.625	25 50 2.500	50 100 5.000	14.06 28.13 1.406
*E. floccosum *(3)	KTZ GRS PPL	0.39- 0.78 3.12-25.00 0.312- 2.500	0.39 6.25 0.625	0.78 25 2.500	1.56 50 5.000	0.585 14.06 1.406

^a^ Values are given as mean of triplicate experiments. The values are reported as μg/mL for KTZ (Ketoconazole ), GRS (greseofulvin) and mg/mL for PPL (*Pogostemon parviflorus *leaf) extracts. ^b^MICs at which 50 and 90% of the isolates in the test panel have been inhibited, respectively.

***T =*** *Trichophyton,* ***M =*** *Microsporum,* ***E =*** *Epidermophyton.*

We also tested *Pogostemon parviflorus *against several strains of *E. floccosum *that produces arthroconidia. These microorganisms survive for a longer time than the other dermatophytes, and as a result constitute an environmental source of contagion, sometimes leading to recurrent outbreaks of dermatophytosis (6). *Trichophyton rubrum *and *T. mentagrophytes*, which are the main cause of athlete’s foot and onichomycoses in human beings, were also tested. Athlete’s foot is the most prevalent superficial infection in the developed world (14) and onichomycoses affects 2%–13% of the population worldwide and up to 30% of groups at high risk, such as elderly and diabetic people (15, 16).

Results of phytochemical screening indicated that the leaf of *Pogostemon parviflorus *contained saponins, reducing sugars, tannins, phenols and proteins, but not glycosides, anthraquinones, alkaloids and flavonoids (Table 3). 

**Table 3:** Phytochemical screening of *Pogostemon parviflorus *leaf extracts

Name of the test carried out	Reagents used	End result
**A. Water extract **		
Starch	I2-KI	+
Tannins	Acidic FeCl3	+
Saponins	H2SO4 + Acetic unhydride	+
Proteins	Millon’s test	+
Anthraquinones	+ Benzene 10% NH4OH	-
Reducing sugars	Benedict’s	+
**B. Alcoholic extract **		
Alkaloids	Mayer’s	-
	Wagner’s	-
	Dragendorff’s	-
Flavonoids	HCl + Mg turnings	-
Glycosides	Benzene+hot ethanol	-

**Figure. 4:**
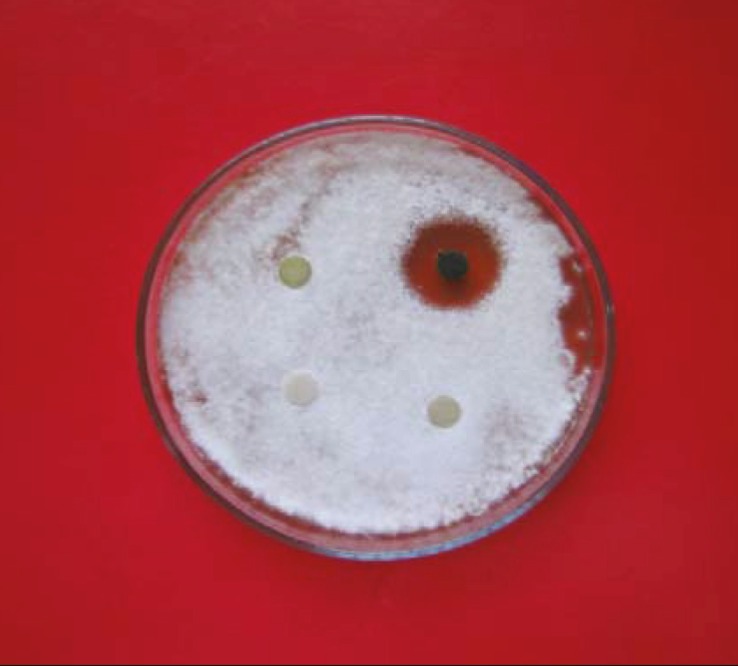
Activity of *Pogostemon parviflorus *extracts made with various organic solvents against *T. mentagrophytes, *using the disk diffusion method

**Figure 5:**
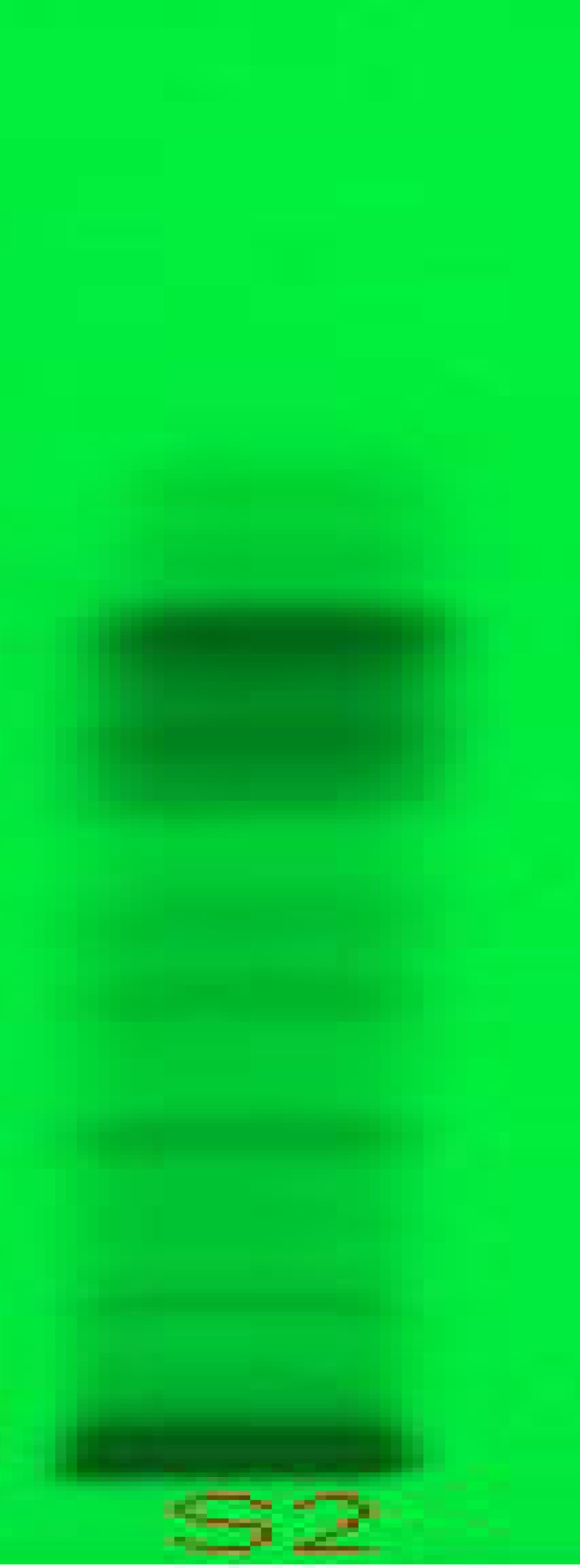
Image at 245 nm: before derivatization

**Figure 6:**
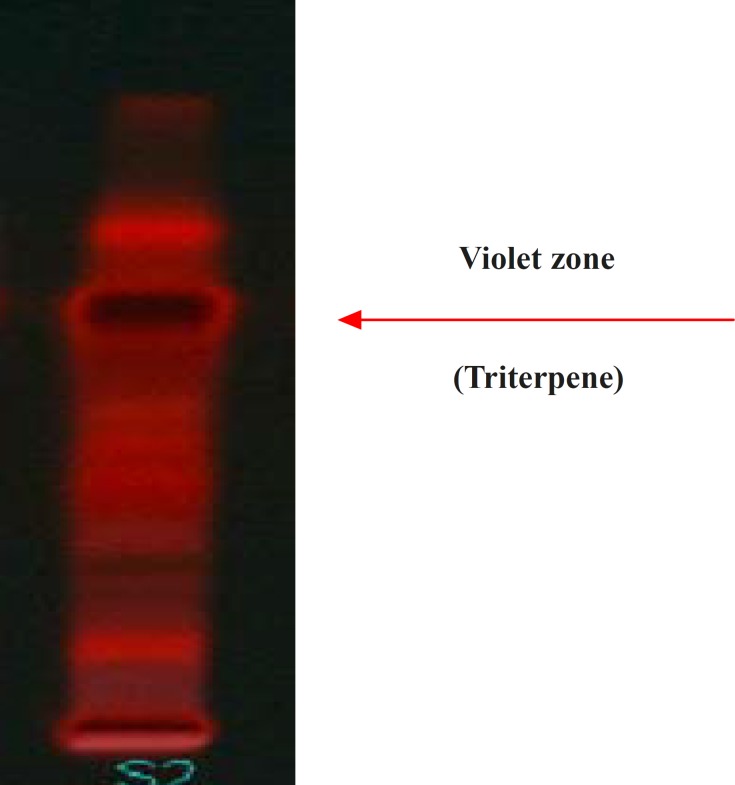
Image at 366 nm: before derivatization

**Figure 7:**
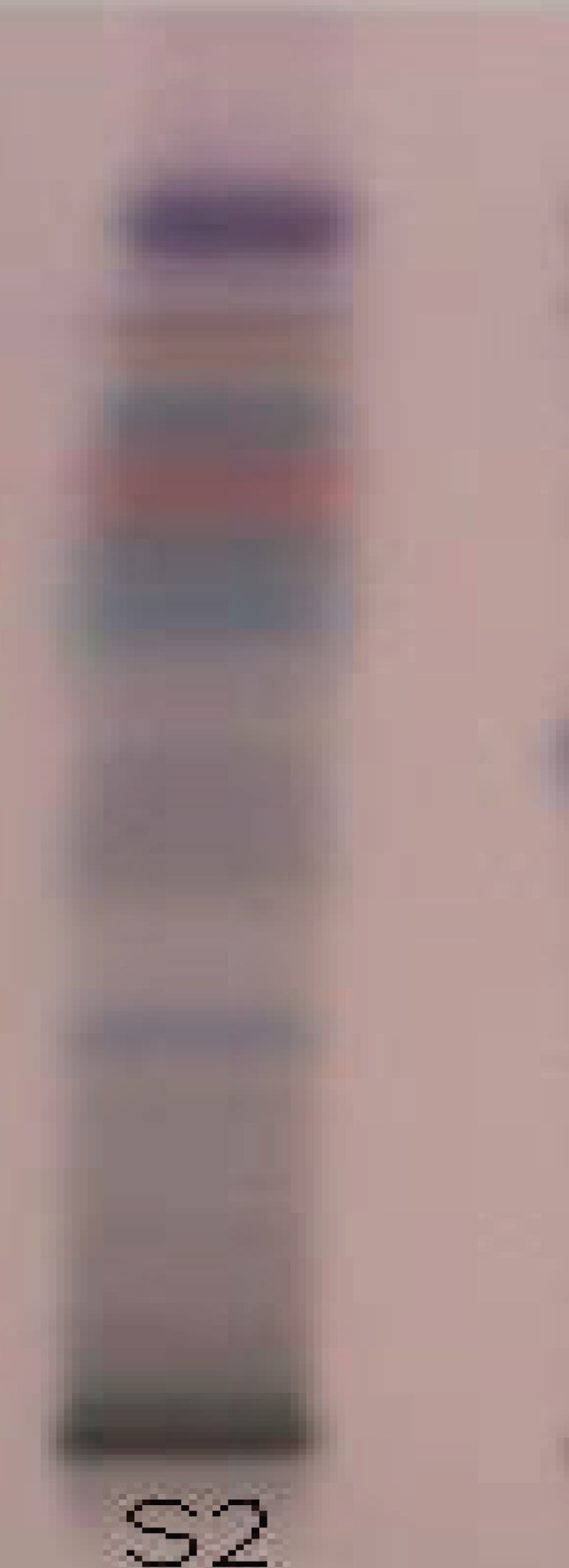
Image at visible light, after derivatization with anisaldehyde sulphuric acid

**Figure 8:**
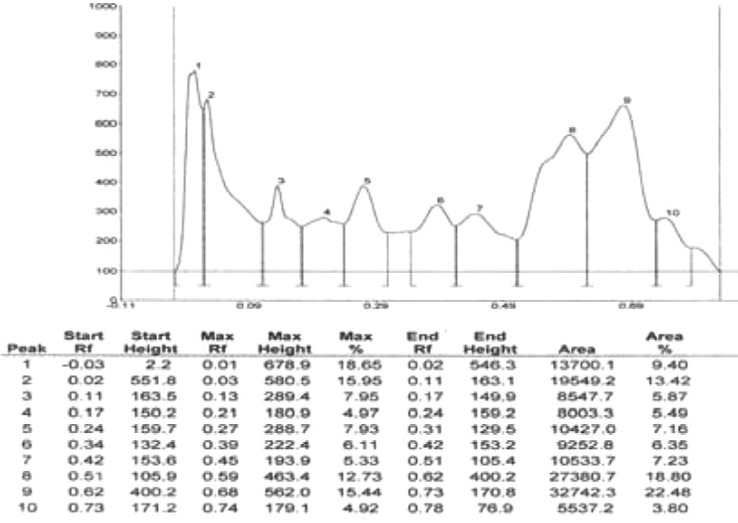
Estimation of triterpenes at 254 nm, before derivatization from *Pogostemon parviflorus *leaf

**Figure 9:**
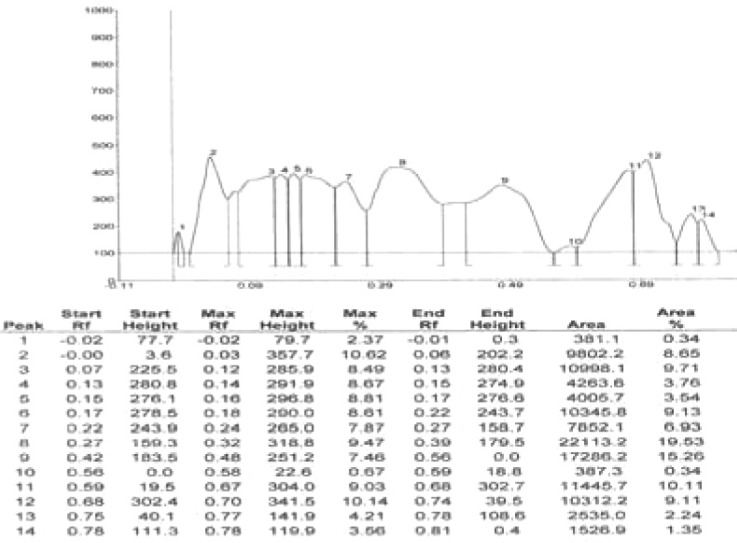
Estimation of triterpenes at 366 nm, after derivatization from *Pogostemon parviflorus *leaf.

Results of HPTLC studies indicated that the ethyl acetate extracts of *Pogostemon parviflorus *leaves (17) contain triterpene, showing 10 uv-absorption peaks in 254 nm, 14 peaks in 366 nm and a violet zone in visible wavelengths after derivatization with anisaldehyde sulphuric acid (Figures 5-9). This compound may account for the anti-dermatophytic activity of this plant. This finding was in agreement with a previous study reporting that the ethanolic extract of *Pogostemon parviflorus *leaf possesses antifungal properties against dermatophytic species isolates (18).

Briefly, based on the results of this study, we can consider the ethanolic extract of *Pogostemon parviflorus *as a new source for developing local antifungal agents. However, further studies are needed to determine the efficacy of active chemical constituents of this plant extract. Toxicological studies must also be performed to ensure the safety of the extract. 
